# Human spinal height growth: a description of normal spine growth patterns and adult spine height prediction from a longitudinal cohort

**DOI:** 10.1007/s43390-024-01011-w

**Published:** 2024-11-25

**Authors:** James O. Sanders, Sarah E. Obudzinski, Lauren E. Karbach, Xing Qiu, Raymond W. Liu

**Affiliations:** 1 Department of Orthopaedics, University of North Carolina at Chapel Hill, Chapel Hill, NC USA; 2 The San Antonio Orthopaedic Group, San Antonio, TX USA; 3 Department of Biostatistics and Computational Biology, University of Rochester, Rochester, NY USA; 4 Department of Orthopaedics, Case Western Reserve University, Cleveland, OH USA

**Keywords:** Spinal growth, Peak height velocity, Adolescent growth spurt, Growth prediction

## Abstract

**Purpose:**

This study describes spinal growth and predicts future growth by standardizing timing relative to the growth spurt.

**Methods:**

From a longitudinal cohort of normal, healthy children followed through their growth, we identified those who completed their growth and compared spinal heights to chronological age and timing relative to the growth spurt. Anthropometrics and radiographs were correlated to identify heights to C1, T1, and S1 using three separate methods with validation performed by comparing to heights predicted by pelvic width. Heights and spinal lengths were normalized to percentages of adult lengths, and multipliers of growth remaining determined for both age and timing relative to PHV_90%_ (peak height velocity defined by achieving 90% of final height) as adult length divided by current spine length. The age at PHV_90%_ is termed Peak Growth Age (PGA)_90%_.

**Results:**

Fifty-four subjects completed their growth at the study terminus (35f, 19 m). We identified multipliers allowing calculations of adult spine length based on the child’s current timing relative to peak growth. At PHV_90%_, children were 90% adult total height and 87% adult spine height. During childhood, spinal growth is 1.55 ± 0.21 cm/yr in girls, 1.14 ± 0.23 cm/yr in boys increasing to 1.75 ± 0.11 cm/yr in girls and 2 ± 0.11 cm/yr in boys during the growth spurt.

**Conclusion:**

This study identifies multipliers of spinal growth determination and identifies their values relative to the adolescent growth spurt timing which is known to be closely related to skeletal maturity. Timing compared to the PGA_90%_ provides reliable predictions of final spine length for both sexes.

## Introduction

Marked spinal deformity progression typically occurs during the growth spurt, and predicting future spine growth is critical to evolving spinal growth modification deformity correction techniques. Current spine growth data derive from very small cross-sectional studies of normal children [1–9]. These cross-sectional studies are problematic because the adolescent growth spurt timing varies between children. Understanding spinal growth during this phase requires longitudinal study identifying where a child is within their growth spurt. A child’s future growth at mid-growth spurt is very similar to other children at mid-growth spurt despite different ages and very different from a same aged child much earlier or later in their growth spurt [10, 11]. Cross-sectional studies cannot demonstrate the true pattern of an individual’s growth spurt because averaging children having different maturities blunts the individual changes. The most referenced source for spinal growth information, DiMeglio [5, 12–16], is based upon data from six thesis inaccessible to review and not well delineated. All other existing studies of spinal height growth are cross-sectional [1–4, 6–9, 17–19].

The Todd collection, funded by the Bolton and Brush Foundations, remains the largest and most complete longitudinal collection of both radiographs and anthropometrics on normal growing children. The study enrolled healthy largely middle class, white children, age 3 months to 14 years at enrollment from 1931 to 1942 with regular anthropometrics and radiographs and is the Greulich and Pyle Atlas [20] source. One of the few available sources of normal growth relative to skeletal maturity*** and impossible to duplicate today because of radiation concerns, it has not been systematically studied using modern longitudinal data analysis techniques.

This study’s purpose is to identify reliable measures of future spinal growth using anthropometrics together with radiographs prior to and through the growth spurt using the findings of Sanders, et al. [10], which identified a uniform pattern of growth in children relative to the timing of the peak height velocity (PHV) and skeletal maturity. Specifically, PHV timing corresponds to 90% of adult height. This maximum growth rate identified at 90% final height is termed the Peak Growth Age 90% (PGA_90%_) and is clearly reflected in skeletal maturity. The correlation between 90% adult height and PHV is significant because 90% adult height can be much more accurately assessed for individual subjects and populations, and represents a cleaner gold standard for determining maturity in research studies. For terminology, we use PGA_90%_ for timing in years relative to the growth peak and PHV for peak height velocity rather than its timing and PHV_90%_ for PHV determined by the velocity at PGA_90%_.

For this study, we used multipliers to describe the height of the spine at a specific time compared to its mature height. If a child has a multiplier of a specific value at a given maturity stage, then the ultimate mature length or height will be the current length multiplied by the multiplier at maturity. The inverse of the multiplier is the proportion of growth completed at that stage. For example, a multiplier of 1.5 means the height will increase by 1.5 times the current height or that the current height is 1/1.5 = 66.7% of mature height.

## Methods

**Fig. 1:**
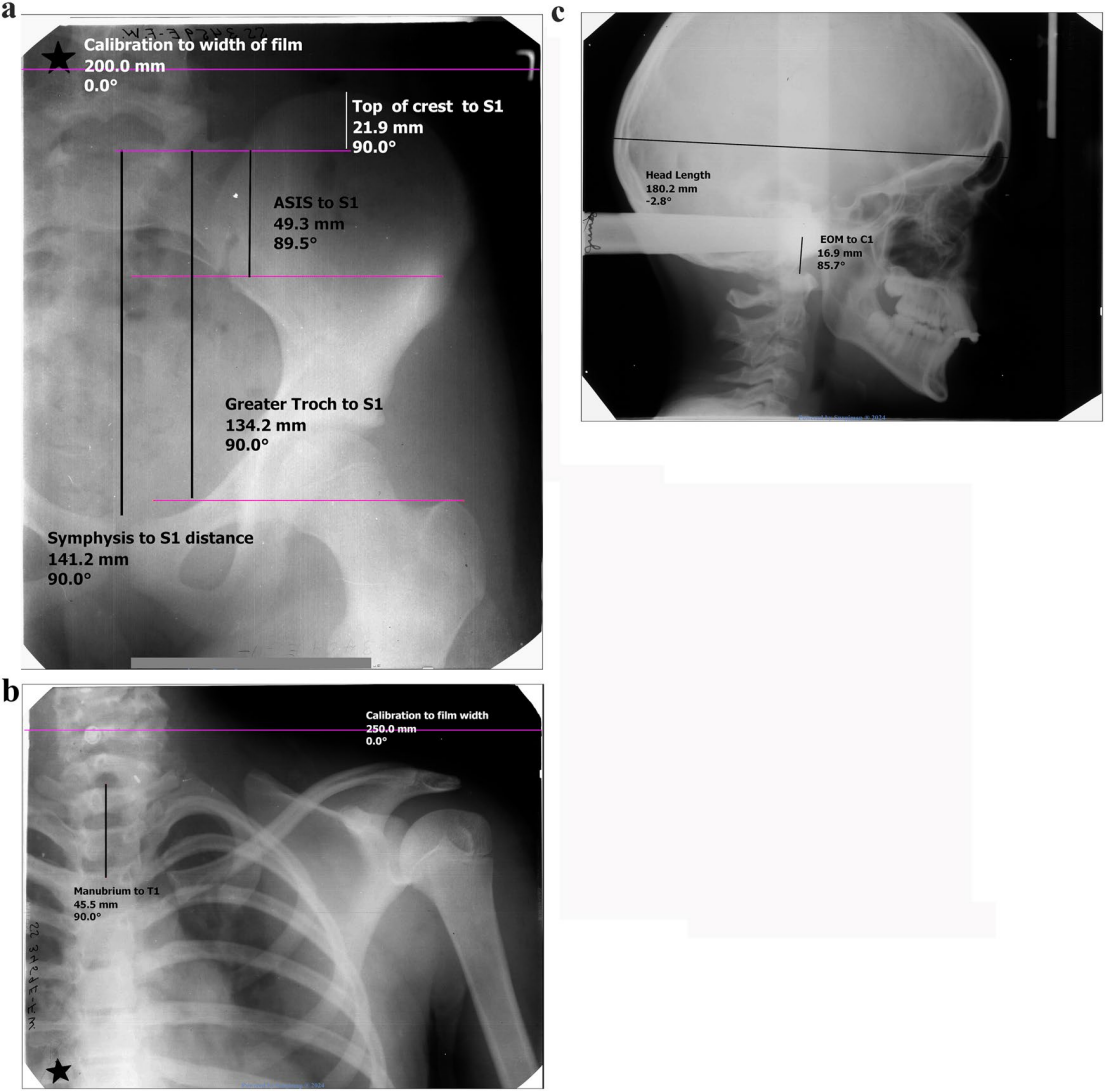
The radiographic measurements evaluating the distances from the pubic symphysis, iliac crest and ASIS to S1 from the left hip (**A**), the distance from the manubrium to T1 on the left shoulder (**B**), and the external auditory meatus to C1on the lateral skull radiographs (**C**)

From the Bolton–Brush Collection, we identified subjects clearly completing growth with criteria of < 1 cm/year increase in standing height at the final visit. The radiographic and anthropometric techniques were described by Simmons and Greulich [21] (Appendix 1). Along with other anthropometrics, subjects had serial standing and sitting heights, heights to the superior symphysis pubis, ASIS, iliac crest, greater trochanter, acromion, sternal notch, and head height (top of head to external auditory meatus). From the radiographs, of the cervical spine, shoulder, and hip which were obtained supine, we identified corresponding points on the anthropometrics and measured the vertical distance to C1, T1, and S1. The top of C1 was identified on the lateral skull, T1 on the left shoulder AP, and S1 on the left hip AP radiographs. Measurements were calibrated to the film dimension for the hip and shoulder images and to the skull length on the skull radiographs (see Fig. 1).

We performed three separate methods of assessing T1-S1 height and, for validation, compared each to pelvic width predictions [22].

1. T1 height as standing height to sternal notch plus radiographic vertical distance from sternal notch to top of T1 and S1height as height to the symphysis pubis, iliac crest or ASIS depending on hip radiograph clarity plus radiographic vertical distance to top of S1.
2. T1-S1 as median percentage of all T1-S1/C1-S1 heights (76.2% ± 1.76%), using top of the head minus the head height as approximate C1 with S1 height the same as in Method 1. Of the 18 subjects with specific skull films, C1 to the external auditory meatus averaged 1.7 cm (± 0.3 cm).
3. T1-S1 as statistical relationship between both C1-S1 and T1-S1 to body height using the regression model of ${\text{BodyHeight}}_{ij}={\text{SpineHeight}}_{ij}\times \widehat{\beta }+{\epsilon }_{ij},$ with $i,j$ representing the *i*th subject and *j*th time point, respectively.

For each time point, current T1-S1 height was determined and compared to the mature height to determine the patient specific multiplier for the specific method. We then compared the spinal heights with timing relative to PGA_90%_ and chronological age. Spline curves were generated to compare the multipliers to PGA_90%_ and age (*GraphPad Prism 10.2.2, 2024 for Windows*). All measurements were made in millimeters either annually or biannually depending upon the child’s age.

For validation, the results were compared both to the stem height (supine ischium to top of head distance) multipliers previously identified by Sanders, et al. [23] and to spine length predicted from pelvic width [7, 24].

### Spine growth velocities

Piecewise linear regression model (*R programming language, version 3.2.0*) was performed using the following phases: childhood as -infinity to −2yrs from PGA_90%_, growth spurt as −2 to 2yrs, terminal growth as 2 to 4yrs, and maturity as 4yrs to infinity. The slopes were used to identify spine growth velocity for each phase.

## Results

Fifty-four subjects completed growth at study terminus (35f, 19 m). First visit age of girls ranged from 2 to 10yrs and boys 7.5–11yrs. PHV timing for girls ranged from 9.7 to 13.4yrs, average 11.3yrs, and boys 11.7–14.3 with average 13.0yrs. Final standing heights ranged from 151 to 175 cm (average 163.4 cm) for girls and 169–183.9 cm (average 177 cm) for boys. For Method 3, identified $\widehat{\beta }$ was 1.378.

Throughout the study time period, subjects’ head heights did not substantially change, indicating cranial height reached maturity at an early age (previously identified by Simmons [21]).

**Fig. 2:**
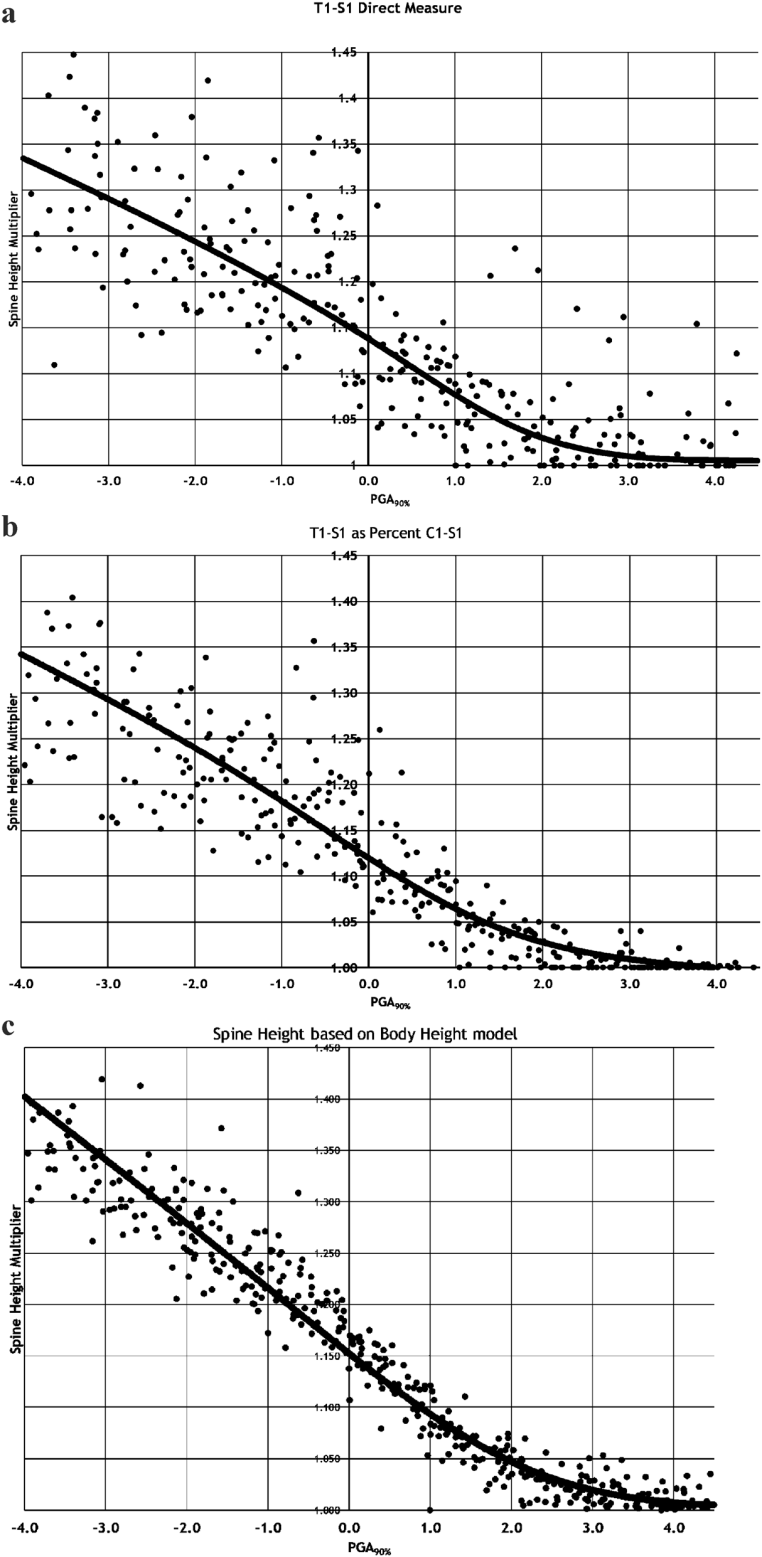
Multiplier plots of each of the three methods compared to PGA90%. 1A Method 1, direct T1-S1 measurement. 1B Method 2, T1-S1 as 76.2% of C1-S1 height. 1C Method 3, based on the statistical relationship between body height and T1-S1 height

Multipliers derived from each method are shown in Fig. 2 and tabulated in Table 1. All methods had similar results but with decreasing scatter going from direct T1-S1 measurement, to fixed percentage, to statistical relationship between body height and T1-S1 with the least variability.

**Table 1:** Multipliers using methods two and three

Timing to PGA90% in years	Spine height multipliers	
	Percent C1-S1	Relative to body height
−4	1.336	1.397
−3.5	1.314	1.366
−3	1.292	1.336
−2.5	1.270	1.305
−2	1.248	1.274
−1.5	1.226	1.243
−1	1.204	1.211
−0.5	1.182	1.179
0	1.160	1.148
0.5	1.138	1.117
1	1.116	1.089
1.5	1.094	1.063
2	1.072	1.042
2.5	1.050	1.026
3	1.028	1.015
3.5	1.006	1.007
4	1.000	1.002
4.5	1.000	1.000

**Fig. 3:**
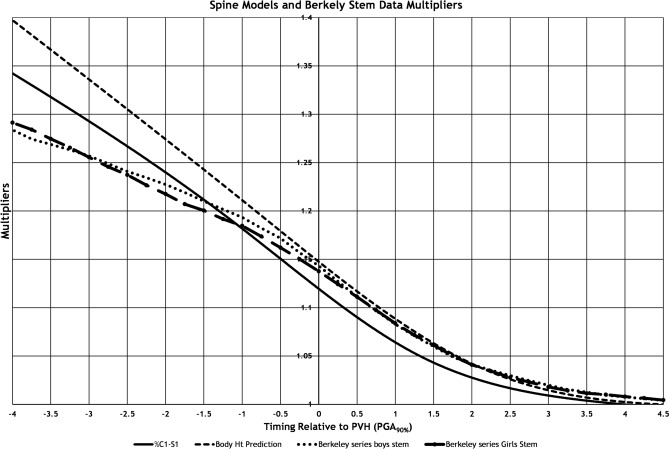
The spline curves of each of the methods and the multipliers from Sanders, et al. [23], of boys’ and girls’ stem length from the Berkeley series

Pelvic width measurements were available for 297 of the 525 potential visits, and the 3 T1-S1 methods were within the 95% confidence interval of the pelvic width to spinal length [7, 24] determinations 97%, 96%, and 98% of the time for methods 1, 2 and 3, respectively. Figure 3 shows the spline curves without individual points compared to the stem length of boys and girls previously determined from the Berkeley series evaluation of children’s limb and height growth [23] which evaluated the stem length (top of head to ischial bottom, essentially sitting height), of every third child born in 1929 Oakland, California.

**Fig. 4:**
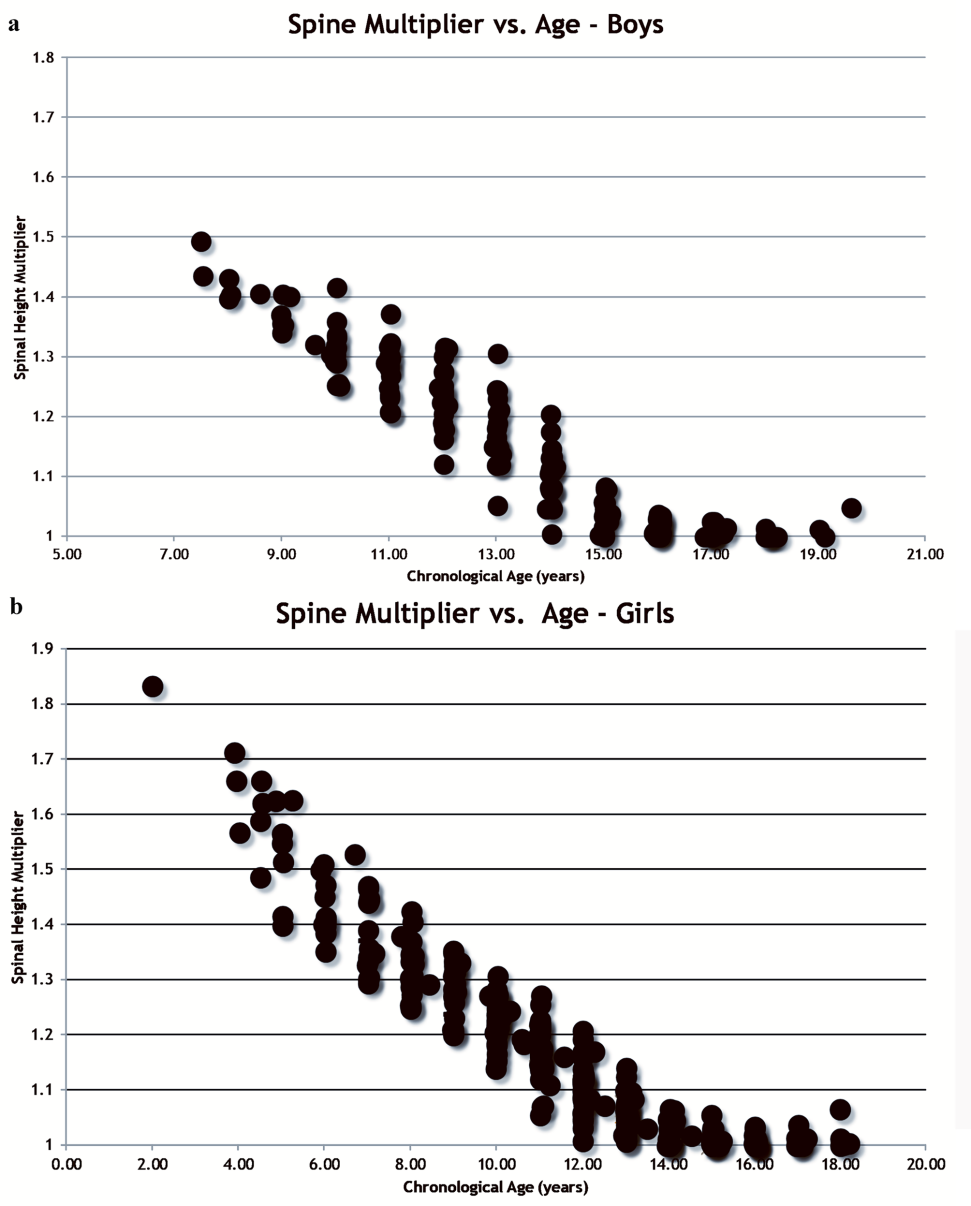
The multipliers of T1-S1 for boys (**A**) and girls (**B**) compared to chronological age using Method 3

Figure 4A, B shows the multipliers for boys and girls separately compared to chronological age. We employed the Wilcoxon signed-rank test on the absolute values of residuals (AVRs) in Figs. 2C, 4A, B to demonstrate that time relative to PGA_90%_ is more strongly associated with the spinal height multiplier than chronological age. Our analysis showed that the median AVR in Fig. 2C is 0.0131, which is significantly lower (*p* < 0.0001) than the pooled median AVR of 0.0206 in Fig. 4A, B. This finding suggests that time to PGA_90%_ is a better predictor of spinal height multiplier than chronological age. In addition, unlike the models using age (Fig. 4A, B), modeling with time to PGA_90%_ (Fig. 2C) does not require separate consideration of two sex groups. However, it is important to note that since the predictors in these two models are not the same, this comparison of residuals should be considered exploratory rather than a formal comparison of models

### Growth rates

Average spinal growth from the entire spine, C1-S1 growth, was 1.3 to 1.6 cm/year during adolescent growth; however, this rate does not delineate the variability in annual growth seen during adolescence. Table 2 provides a detailed summary of T1-S1 growth velocities for boys and girls, stratified by time intervals relative to PGA_90%,_ which allows for more accurate predictions of spinal growth in pediatric populations across different age ranges."

**Table 2:** Spinal growth velocities for boys and girls in cm/yr

	Girls	Boys
≤ −2 PGA_90%_	1.55 (0.212, 1.25)	1.14 (0.231, 1.01)
−2 to + 2 yrs PGA_90%_	1.75 (0.11, 0.65)	2 (0.114, 0.50)
+ 2 to + 4 yrs PGA_90%_	0.9 (0.114, 0.672)	0.881 (0.113, 0.49)
≥ 4yrs PGA_90%_	0.0474 (0.23, 1.36)	−0.12 (0.222, 0.97)

Mean annual growth rate (in cm/year) for T1-S1

Standard error of the mean are reported as the first value in parentheses and standard deviations as the second

During the slower preadolescent growth, rates were 1.5 cm/yr in girls and 1.14 cm/yr in boys, increasing to 1.75 cm/yr in girls and 2 cm/yr in boys from −2yrs to + 2yrs PGA_90%_. From 2 to 4 yrs, growth slows to 0.9 cm/year and then is negligible by 4–4.5 years PGA_90%._

## Discussion

Sanders, et al. [10] previously found PGA_90%_ a useful maturity timing measure reflected in skeletal maturity. As children reach adolescence, their percentage growth remaining is equivalent for both sexes relative to PGA_90%._ Children grow very similarly beginning at about 85% of final height, reaching PHV at 90% final height and growth completion 4–4.5yrs afterward. Growth prior to the growth spurt determines height entering the spurt, which then determines final height. Although PGA_90%_ cannot be identified for children except via its correlates in a clinical setting, it is a powerful gold standard to investigate normal child growth and skeletal maturity. Skeletal maturity is closely related to PGA_90% _[10].

While each method identified spinal lengths consistent with Gold, et al. [24], they were different. Direct T1-S1 measurement was the least consistent which appears to be unreliability of using shoulder radiographs to determine sternal notch -T1 which was susceptible to radiographic positioning. The second method of using a consistent percentage of top of head minus head height to S1 provided much more reliability likely because head radiographs compared to top of head measurements were much more consistent than ***Method 1. This study used the average of all the direct T1-S1/C1-S1 percentages which exhibited no difference over time. The determined ratio is consistent with a detailed cadaveric study by Jason, et al. [25]. The deficiency with Method 2 is that the series changed techniques in 1941–2 with anthropometric measurements to the hips using the greater trochanter rather than the symphysis, crest or ASIS which were more variable and likely underestimated mature spine height. The third method using statistical T1-S1 relationship body height had the lowest variability and allowed use of all the subject measurements.

The three methods generated consistent but slightly different multipliers. The direct technique had by far the most scatter. Method 2 had lower multipliers than Method 3. Figure 3 compares the results of Methods 2 and 3 with prior work by Sanders, et al. [23], demonstrating stem length multipliers from the Berkeley series [23]. Method 2 had the lowest multipliers, the Berkeley boys and girls stem length next highest, and Method 3 the highest. This makes sense as the Berkley stem length included the head which was not growing and Method 3 also included all the measurements where growth had clearly stopped while this was less consistent for Method 2 because of the 1941–2 measurement change. We recommend considering Method 2 the lower limit and Method 3 the higher limit of the multiplier placing greater confidence in Method 3.

This study identifies several important attributes of spinal growth before and during adolescence. First, it quantifies the spine is between 84 and 88% (inverse of the multiplier at PGA_90%_) of its final height at the PHV compared to 90% for overall height. Second, this study affirms the concept of using multipliers for spinal growth. The concept of the multiplier, first described by Bayley using the Berkeley study [26–28], and popularized by Paley and co-investigators [29–35], eliminates determining percentiles since taller children will grow proportionally the same but absolutely more than smaller but similarly mature children. The multiplier accurately describes future periadolescent spinal growth and is much more closely related to the timing of the adolescent growth spurt as determined by the PGA_90%_ and thence to skeletal maturity rather than chronological age. As shown in Fig. 4A, B, especially when compared with Fig. 2C, age clearly has a great deal of variability compared to growth remaining. Age can be a reasonable multiplier estimator in childhood prior to about age 7.5 in girls and 10.5 in boys of future growth, but fails once the child approaches their growth spurt because of the variability in when children enter their growth spurts [23], while PGA_90%_ with its relation to skeletal maturity is superior to and improves throughout the growth spurt.

Third, this study, being longitudinal, quantifies the rapidity of growth before and during the growth spurt. Prior cross-sectional studies could not identify these peaks because design blunted the rapid changes by averaging across subjects. DiMeglio [14, 15] identified growth of the spine as 1.2 cm/year between ages 5–10 and 1.8 cm/year between ages 10 and skeletal maturity. This is similar to our findings of the preadolescent 1.55 cm/yr. in girls and 1.14 cm/yr. in boys and growth spurt velocities of 1.75 cm/yr. in girls and 2 cm/yr. in boys. Unlike DiMeglio, our results indicate that these velocities are tied to the growth spurt rather than specific chronological ages.

### Segmental spine growth

While it is not possible from our study to directly describe individual spinal vertebral growth, it is possible, by extrapolation from cross-sectional studies [36] showing that during adolescence, the thoracic spine composes 63% and the lumbar spine 27% of T1-S1 height, to estimate segment growth. From this, the growth of each vertebral body, thoracic, and lumbar is the same 0.8 mm/segment/year in preadolescent girls, 0.6 mm/segment/year in preadolescent boys and increasing to 1 mm/segment/year in adolescent girls and 1.1 mm/segment/year in adolescent boys. Our preadolescent values are similar to Winter’s estimate of 0.7 mm/segment/year [37], but more rapid during the growth spurt. Precise individual spinal level growth requires further study.

A potential limitation is that the measurements are derived from radiographic correlates to anthropometric measurements with inherent variability as seen in the scatter plots. Magnification and radiographic parallax also create potential errors, but the consistency with Gold, et al.’s [24] pelvic width to spinal length measurements and the consistency with human stem lengths [23] is very reassuring.

Another potential limitation is we only evaluated longitudinal height and did not include sagittal alignment changes. Some of this discrepancy is likely minimized since stem lengths are obtained supine, flattening both the thoracic kyphosis and lumbar lordosis. We were also unable to assess the growth of individual vertebrae or discs nor their contribution to spine length. Detailed cross-sectional data using modern imaging to provide accurate coronal and sagittal lengths are essential to enhance our knowledge of the various segments.

Because this study was conducted on healthy, middle class, primarily white children, whether is applicable to current children depends on its validity for other populations of children since there is evidence of some maturational differences between the 1930s and today [38]. Across all ages compared to the most recent NHANES (the National Health and Nutrition Examination Survey run by the CDC providing the standard data for growth charts) data [39], the Brush series average and median percentiles were, respectively, 56.5 percentile (ptl) and 60.5 ptl for girls and 60.9 ptl and 62.8 ptl for boys demonstrating equivalence between the Brush and modern populations. Repeating this study for modern children is not possible because, while it is feasible to follow children’s anthropometrics longitudinally [40], regular skeletal maturity radiographs can no longer be obtained in longitudinal studies of healthy children because of radiation concerns. Sanders, et al. [10], have delineated the evidence from other studies that despite some minor differences, the growth patterns relative to skeletal maturity and relative growth as reflected in the multipliers have remained constant across time and geography providing good evidence that the relative values of growth remaining are fundamental to human biology. It remains important to determine whether this is similar in children with various types of spinal deformity since correcting deformity by growth modification requires growth predictability.

In summary, we have identified a common pattern of spinal growth in healthy children before and during the adolescent growth spurt which is predictable based upon where the child is within in their growth spurt. The spine has more growth remaining at the beginning and peak of the growth spurt than the axial skeleton, and we provide multipliers of future spinal growth and velocities which can potentially be used to predict results of spinal growth modification in normal children. To predict future spine growth, we recommend using multipliers based on timing relative to the PGA_90%_ in Table 2 with those of the body height method as the upper and percent C1-S1 method as the lower limit.

## Data Availability

Data from this study is not publically available and is the property of Case Western University but may be obtained by specific request.

## Appendix

### Anthropometric measurement methods

These methods are extracted from Simmons^22^.

The greater number of all measurements reported here were made by two observers, Dr. C. C. Francis and Miss R. Locher. In the early years of the study, some measuring was done by Drs. T. Wingate Todd, W. M. Krogman, and T. T. Zuck, and in the last 2 years, the measurements were taken by Misses I. Pyle and I. Lieb.

Measurements were made of the left side of children, dressed in indoor clothing, shoes removed. Most of the instruments used were built in the shop of the Laboratory of Anatomy, Western Reserve University, and include the common type of spreading caliper of 300 mm; span, the segmented anthropometer of Martin design, Todd’s Reserve head-spanner, a full-length measuring table similar to the design of Thompson, and a steel millimeter tape. The measuring techniques observed until June, 1941, were as follows (the current investigators have included only those measurements used in this investigation of spinal growth.):

Standing height. The subject stood free, not against a wall. Height was measured with the anthropometric rod from vertex to floor. Care was taken to keep the shaft of the anthropometer in vertical position. The lower cross-arm of the anthropometer was brought to the vertex without using pressure.

Horizontal length. The subject lay on his/her back on the steel measuring table which is covered with a hard sponge rubber pad, his feet flush with the end plate which bears the foot of an anthropometer with a single cross-arm. The shaft of the anthropometer is parallel to the horizontal surface of the table and perpendicular to the end board. The measurement was made from vertex to heel.

Sitting height. The subject sat on a flat surfaced bench, his spine and neck as erect as possible, his head in the Frankfort plane. The foot of the anthropometer rested on the flat surface of the bench. The measurement was made from vertex to bench.

Stem length. The subject lay on his back on the measuring table, his buttocks against the end plate of the table, his knees together and flexed. The measurement was made from vertex to buttocks.

Cristal height. The measurement was made from the highest point of the iliac crest (anterior) to the floor.

Symphysial height. The measurement was made from the superior border in the midline of the symphysis pubis to the floor.

Anterior iliac spinous height. The measurement was made from the highest point on the curve of the anterior superior spine of the ilium to the floor.

Acromial height. The measurement was made from the upper margin of the most lateral portion of the acromion to the floor.

Suprasternal height. The measurement was made from the superior border of the suprasternal notch to the floor.

Head height (auricular). The measurement was made with Todd’s Reserve head spanner. The two side rods of the instrument were firmly set in the external auditory canals and the vertical measuring scale was in contact with the scalp at the vertex. The measurement taken, therefore, was the distance from auditory meatus to vertex.

All linear measurements were recorded to the nearest millimeter.
